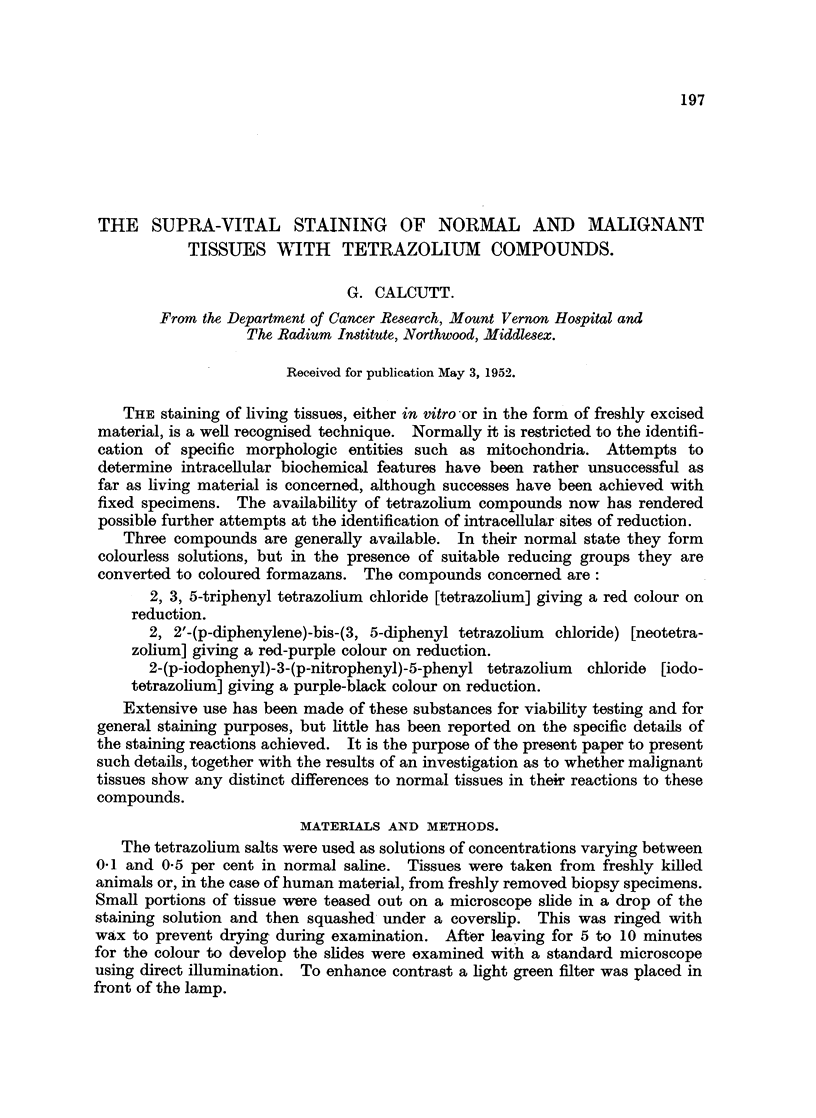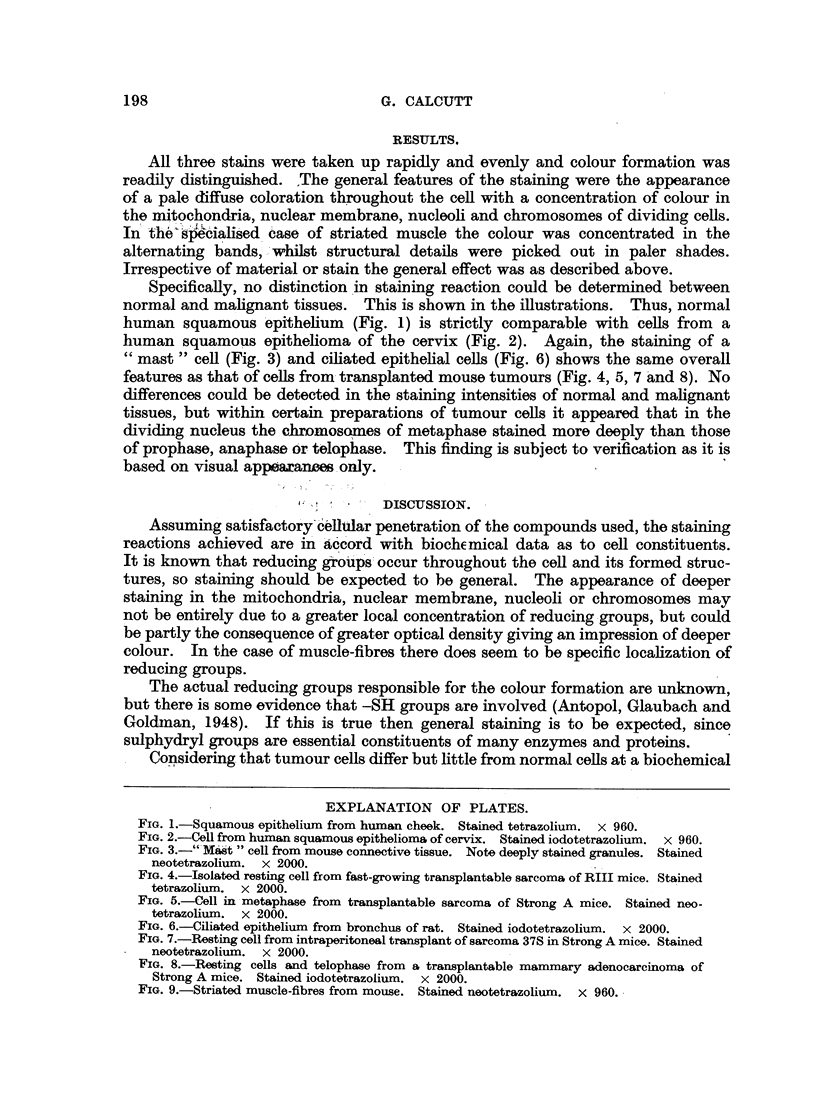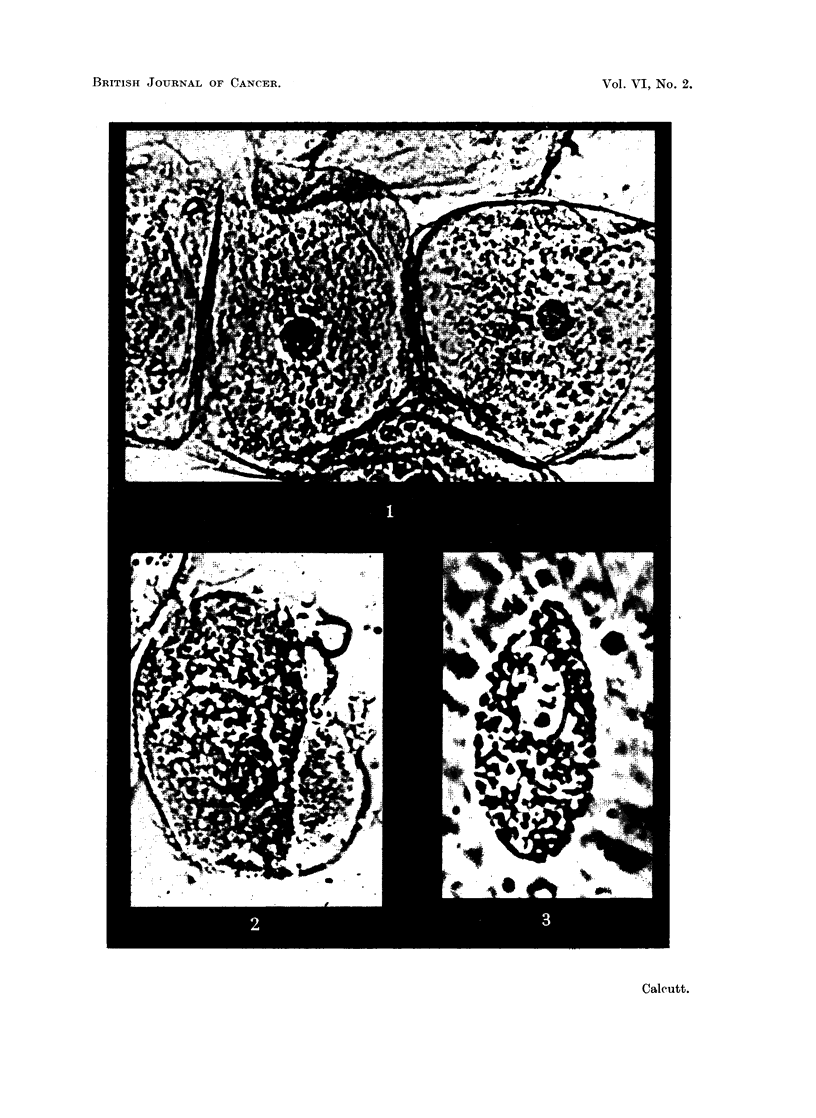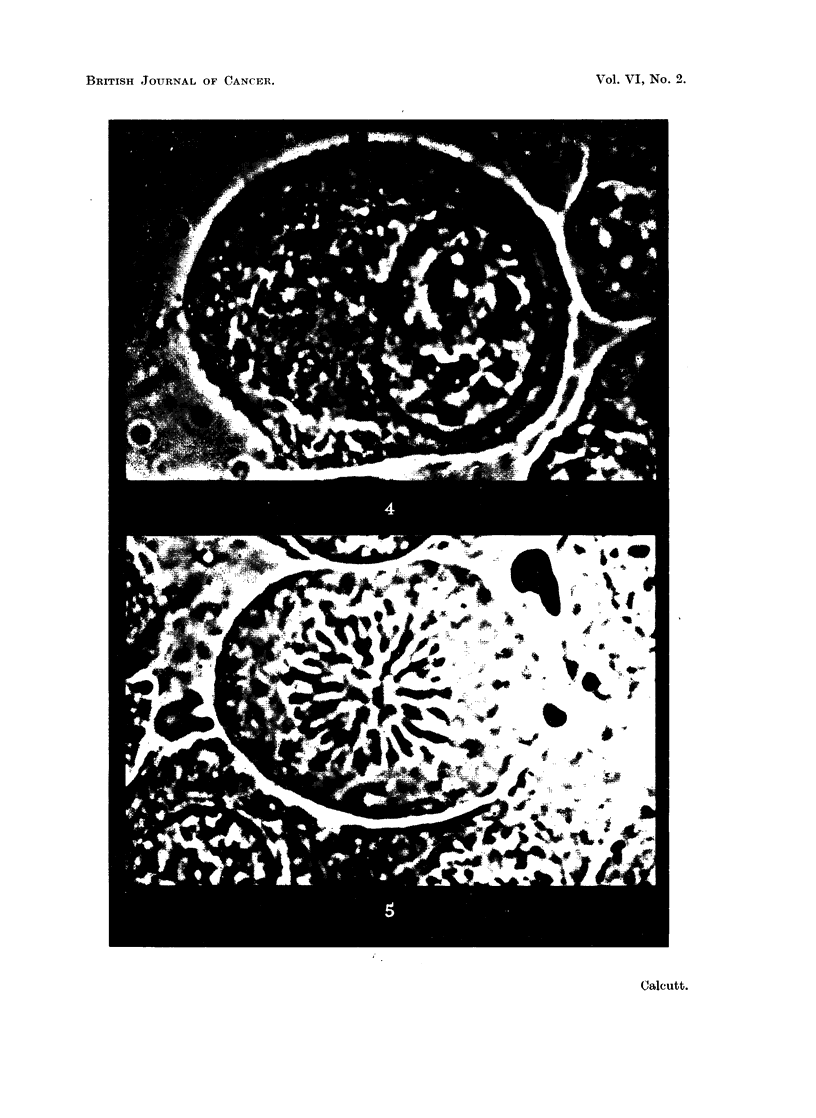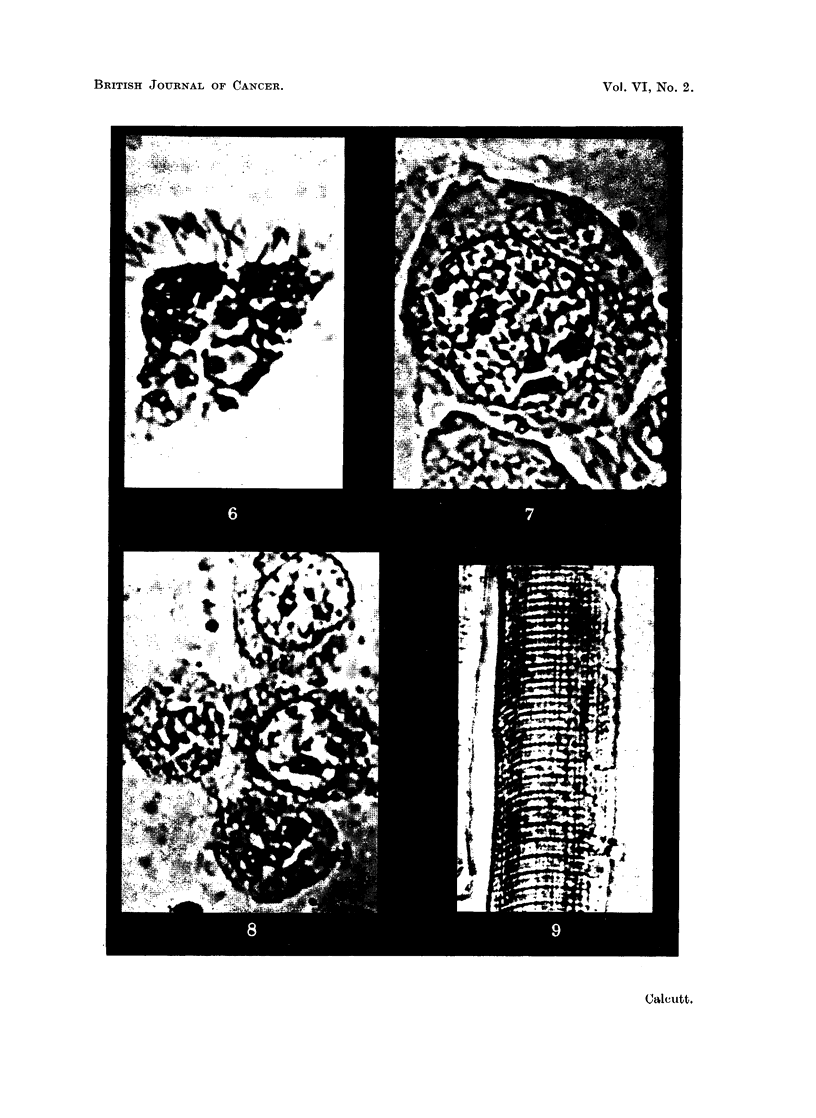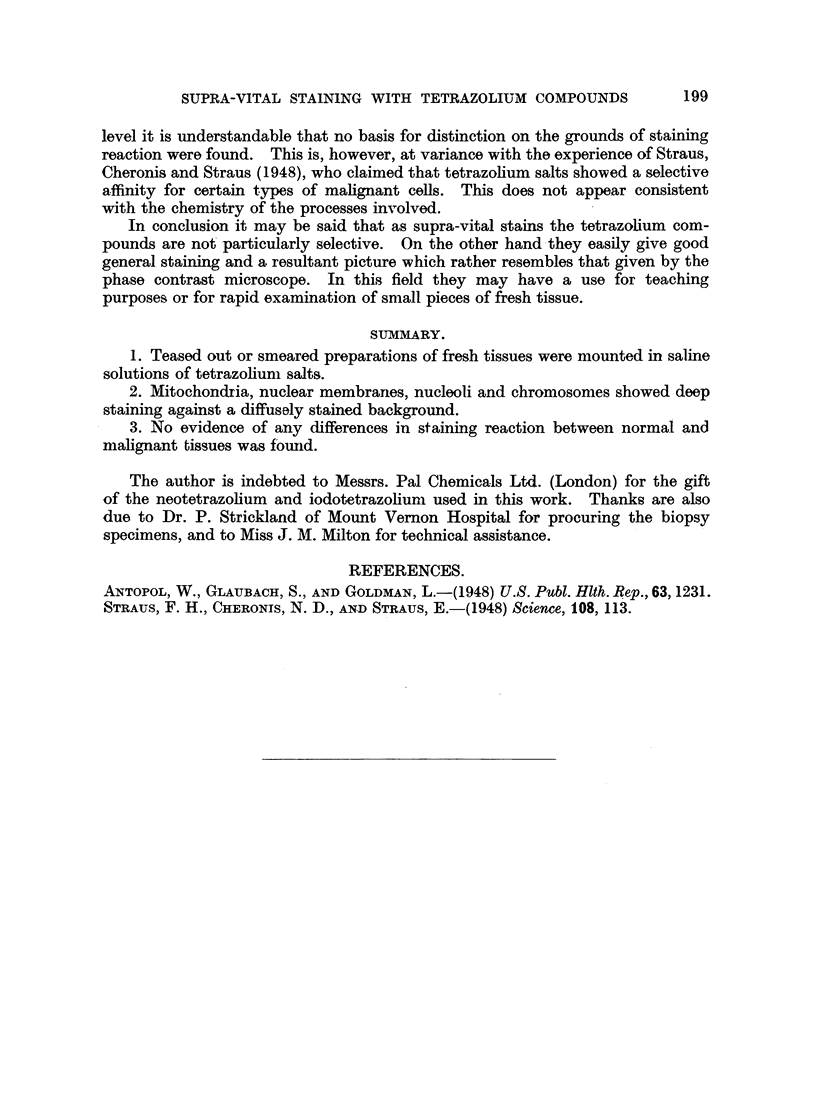# The Supra-vital Staining of Normal and Malignant Tissues with Tetrazolium Compounds

**DOI:** 10.1038/bjc.1952.22

**Published:** 1952-06

**Authors:** G. Calcutt

## Abstract

**Images:**


### THE SUPRA-VITAL STAINING OF NORMAL AND MALIGNANT TISSUES WITH TETRAZOLIUM COMPOUNDS.

G. CALCUTT.

*From the Department of Cancer Research, Mount Vernon Hospital and The Radium Institute, Northwood, Middlesex.*

Received for publication May 3, 1952.

THE staining of living tissues, either *in vitro* or in the form of freshly excised material, is a well recognised technique. Normally it is restricted to the identification of specific morphologic entities such as mitochondria. Attempts to determine intracellular biochemical features have been rather unsuccessful as far as living material is concerned, although successes have been achieved with fixed specimens. The availability of tetrazolium compounds now has rendered possible further attempts at the identification of intracellular sites of reduction.

Three compounds are generally available. In their normal state they form colourless solutions, but in the presence of suitable reducing groups they are converted to coloured formazans. The compounds concerned are :

2, 3, 5-triphenyl tetrazolium chloride [tetrazolium] giving a red colour on reduction.

2, 2′-(p-diphenylene)-bis-(3, 5-diphenyl tetrazolium chloride) [neotetrazolium] giving a red-purple colour on reduction.

2-(p-iodophenyl)-3-(p-nitrophenyl)-5-phenyl tetrazolium chloride [iodotetrazolium] giving a purple-black colour on reduction.

Extensive use has been made of these substances for viability testing and for general staining purposes, but little has been reported on the specific details of the staining reactions achieved. It is the purpose of the present paper to present such details, together with the results of an investigation as to whether malignant tissues show any distinct differences to normal tissues in their reactions to these compounds.

### MATERIALS AND METHODS.

The tetrazolium salts were used as solutions of concentrations varying between 0·1 and 0·5 per cent in normal saline. Tissues were taken from freshly killed animals or, in the case of human material, from freshly removed biopsy specimens. Small portions of tissue were teased out on a microscope slide in a drop of the staining solution and then squashed under a coverslip. This was ringed with wax to prevent drying during examination. After leaving for 5 to 10 minutes for the colour to develop the slides were examined with a standard microscope using direct illumination. To enhance contrast a light green filter was placed in front of the lamp.

### RESULTS.

All three stains were taken up rapidly and evenly and colour formation was readily distinguished. The general features of the staining were the appearance of a pale diffuse coloration throughout the cell with a concentration of colour in the mitochondria, nuclear membrane, nucleoli and chromosomes of dividing cells. In the specialised case of striated muscle the colour was concentrated in the alternating bands, whilst structural details were picked out in paler shades. Irrespective of material or stain the general effect was as described above.

Specifically, no distinction in staining reaction could be determined between normal and malignant tissues. This is shown in the illustrations. Thus, normal human squamous epithelium (Fig. 1) is strictly comparable with cells from a human squamous epithelioma of the cervix (Fig. 2). Again, the staining of a " mast " cell (Fig. 3) and ciliated epithelial cells (Fig. 6) shows the same overall features as that of cells from transplanted mouse tumours (Fig. 4, 5, 7 and 8). No differences could be detected in the staining intensities of normal and malignant tissues, but within certain preparations of tumour cells it appeared that in the dividing nucleus the chromosomes of metaphase stained more deeply than those of prophase, anaphase or telophase. This finding is subject to verification as it is based on visual appearances only.

### DISCUSSION.

Assuming satisfactory cellular penetration of the compounds used, the staining reactions achieved are in accord with biochemical data as to cell constituents. It is known that reducing groups occur throughout the cell and its formed structures, so staining should be expected to be general. The appearance of deeper staining in the mitochondria, nuclear membrane, nucleoli or chromosomes may not be entirely due to a greater local concentration of reducing groups, but could be partly the consequence of greater optical density giving an impression of deeper colour. In the case of muscle-fibres there does seem to be specific localization of reducing groups.

The actual reducing groups responsible for the colour formation are unknown, but there is some evidence that –SH groups are involved (Antopol, Glaubach and Goldman, 1948). If this is true then general staining is to be expected, since sulphydryl groups are essential constituents of many enzymes and proteins.

Considering that tumour cells differ but little from normal cells at a biochemical

---

### EXPLANATION OF PLATES.

Fig. 1.—Squamous epithelium from human cheek. Stained tetrazolium. × 960.

Fig. 2.—Cell from human squamous epithelioma of cervix. Stained iodotetrazolium. × 960.

Fig. 3.—" Mast " cell from mouse connective tissue. Note deeply stained granules. Stained neotetrazolium. × 2000.

Fig. 4.—Isolated resting cell from fast-growing transplantable sarcoma of RIII mice. Stained tetrazolium. × 2000.

Fig. 5.—Cell in metaphase from transplantable sarcoma of Strong A mice. Stained neotetrazolium. × 2000.

Fig. 6.—Ciliated epithelium from bronchus of rat. Stained iodotetrazolium. × 2000.

Fig. 7.—Resting cell from intraperitoneal transplant of sarcoma 37S in Strong A mice. Stained neotetrazolium. × 2000.

Fig. 8.—Resting cells and telophase from a transplantable mammary adenocarcinoma of Strong A mice. Stained iodotetrazolium. × 2000.

Fig. 9.—Striated muscle-fibres from mouse. Stained neotetrazolium. × 960.

1

2

3

Calcutt.

4

5

6

7

8

9

Calcutt.

level it is understandable that no basis for distinction on the grounds of staining reaction were found. This is, however, at variance with the experience of Straus, Cheronis and Straus (1948), who claimed that tetrazolium salts showed a selective affinity for certain types of malignant cells. This does not appear consistent with the chemistry of the processes involved.

In conclusion it may be said that as supra-vital stains the tetrazolium compounds are not particularly selective. On the other hand they easily give good general staining and a resultant picture which rather resembles that given by the phase contrast microscope. In this field they may have a use for teaching purposes or for rapid examination of small pieces of fresh tissue.

### SUMMARY.

1. Teased out or smeared preparations of fresh tissues were mounted in saline solutions of tetrazolium salts.

2. Mitochondria, nuclear membranes, nucleoli and chromosomes showed deep staining against a diffusely stained background.

3. No evidence of any differences in staining reaction between normal and malignant tissues was found.

The author is indebted to Messrs. Pal Chemicals Ltd. (London) for the gift of the neotetrazolium and iodotetrazolium used in this work. Thanks are also due to Dr. P. Strickland of Mount Vernon Hospital for procuring the biopsy specimens, and to Miss J. M. Milton for technical assistance.

### REFERENCES.

Antopol, W., Glaubach, S., and Goldman, L.—(1948) *U.S. Publ. Hlth. Rep.*, **63**, 1231.

Straus, F. H., Cheronis, N. D., and Straus, E.—(1948) *Science*, **108**, 113.